# Broad-spectrum neutralization of avian influenza viruses by sialylated human milk oligosaccharides: *in vivo* assessment of 3′-sialyllactose against H9N2 in chickens

**DOI:** 10.1038/s41598-018-20955-4

**Published:** 2018-02-07

**Authors:** Ramesh Prasad Pandey, Dae Hee Kim, Jinsuk Woo, Jaeyoung Song, Sang Ho Jang, Joon Bae Kim, Kwang Myun Cheong, Jin Sik Oh, Jae Kyung Sohng

**Affiliations:** 1 Institute of Biomolecule Reconstruction, Department of BT-Convergent Pharmaceutical Engineering and Department of Life Science and Biochemical Engineering, Sun Moon University, 70 Sunmoon-ro 221, Tangjeong-myeon, Asan-si, Chungnam 31460 Korea; 2 GeneChem Inc., 59-5 Jang-dong, Yuseong-gu, Daejeon, 305-343 Korea; 3 Animal and Plant Quarantine Agency (QIA) 175 Anynag-ro Manan-gu, Anyang-si, Gyeonggi-do 430-757 Korea; 4 Median Diagnostics Inc., 878, Sunhwan-daero, Dongnae-myeon, Chuncheon-si, Gangwon-do Korea

## Abstract

Two sialylated human milk oligosaccharides (SHMOs) 3′-sialyllactose (3′-SL) and 6′-sialyllactose (6′-SL) were accessed for their possible antiviral activity against six different subtypes of thirteen avian influenza (AI) viruses *in vitro*. 3′-SL exhibited promising antiviral activity against almost all subtypes of tested AI viruses in hemagglutination inhibition assay, whereas 6′-SL showed activity against few selected H1N1, H1N2, and H3N2 subtype strains. 3′-SL has minimum inhibitory concentration values of 15.62 mM or less in more than half of the viruses examined. 3′-SL also showed effective inactivation of H9N2 Korea isolate (A/Chicken/Korea/MS96/1996) at 12.5 mM concentration in Madin Darby Canine Kidney (MDCK) cell line. Thus, 3′-SL was further studied for *in vivo* study against H9N2 virus in pathogen free chicken experiment models. *In vivo* study exhibited improved clinical symptoms on H9N2 infected chickens when treated with 3′-SL. Moreover, treating chickens with 3′-SL resulted in complete elimination of H9N2 viruses within 24 h of virus infection (0.8 HAU of H9N2). Indirect ELISA assay confirmed complete wash-out of H9N2 viruses from the colon after neutralization by 3′-SL without entering the blood stream. These *in vivo* results open up possible applications of 3′-SL for the prevention of AI virus infections in birds by a simple cleansing mechanism.

## Introduction

In recent years, there have been a number of handwringing disease outbreaks worldwide. Most of these outbreaks have been viral infections. For example, global issues that emerged in 2015, 2016, and 2017 included several avian influenza (AI) A H7N9 and H5N6 viruses outbreaks in China; Ebola virus outbreak in West Africa; Zika virus infection in Mexico, Central and South America; multiple Middle East Respiratory Syndrome coronavirus (MERS-CoV) outbreaks in Korea, Saudi Arabia, Qatar, Lebanon, and United Arab Emirates; vaccine-derived poliovirus infections in Myanmar and the Lao People’s Democratic Republic, and Hepatitis A outbreaks in European region and the Americas^1^. These outbreaks brought tremendous causalities, financial burdens, and threats to global health security worldwide. In addition, the world is currently facing the risk of exposure to a number of seasonal and pandemic influenza virus infections.

There are growing numbers of vaccines and antiviral drugs to control the spread of viral outbreaks, many of them are under clinical trials and under approval from U.S. food and drug administration^2–5^. Unfortunately, the existing vaccines are unable to keep up with the mutation rates of viruses^6–8^. It also takes several years to develop vaccine for newly emerged viruses. At the same time, viruses are developing resistance to the currently used drugs, and new drugs need to be developed for new viruses^4,9^. Hence, there is no immediate response drug to the newly emerging virus infections/outbreaks. To address this problem, there is an exigent need for the development of a new paradigm preventive and therapeutic agent to control the immediate spread of viral outbreaks.

In this paper, we first accessed the effectiveness of two enzymatically synthesized sialylated human milk oligosaccharides (SHMOs) 3′-sialyllactose (3′-SL) and 6′-sialyllactose (6′-SL) in the prevention of viral infection using different classes of viral strains in an *in vitro* haemagglutination inhibition (HI) assay. We applied the selected 3′-SL derivative to *in vivo* experiments in pathogen free chickens as model animals for the prevention and treatment of potential pandemic bird flu influenza virus (H9N2) infection, one of the viruses tested. Influenza viruses, particularly influenza A, are the causative agents of seasonal epidemics, causing serious public health problems with 3 to 5 million cases of severe illness, and about 250,000 to 500,000 deaths every year worldwide^10^. Likewise, AI viruses, unlikely to infect humans except few strains such as H5N1 and H7N9, infect birds including poultry, and create severe economic burdens on local and global economic and international trade^10^. H9N2 is one of the highly prevalent AI virus subtypes with wide distribution around the world, since its first detection in Wisconsin in 1966^11^. H9N2 is not only infectious to birds, but has also recently been found to be transmissible to humans^12^, and other animals, such as pigs^13^. In poultry, it causes acute respiratory tract infections, resulting in a drop in egg production, and increased mortality in the field when co-infected with other pathogens^14,15^. The unprecedented rate of spread of H9N2 among egg-laying hens and other poultry in South Korea raised poultry and poultry products’ prices sharply higher. According to the recent news from ministry of agriculture, food and rural affairs, S. Korea culled nearly 38 million farm birds within November 2016 to June 2017, more than a fifth of its total poultry population. More recently, the country is facing severe outbreak of another highly pathogenic AI H5N8 strain, raising country’s bird flu alert to its highest level ‘grave level’ while banning the transportation of poultry and bird farmers nationwide.

Adhesion of pathogens to the host cell surface is a prerequisite for the majority of infections. Prevention of pathogen attachment to the host cell or detachment of adherent pathogen blocks the infectious agent from interaction with the host cell, and subsequent internalization and delivery of virulence factors, and access to the nutrients and evasion of the host immune system. This leads to simple elimination of pathogens from the host by a cleansing mechanism^16^. Bacterial adhesins or lectins are proteins or polysaccharides that are at the surface of the cells, and bind specifically to certain sugar moieties present in the host cell surface^17,18^. Influenza viruses also utilize saccharides binding protein such as haemagglutinin (HA) for adhesion to the host cell. AI virus HA preferably binds to 3′-SL molecules prior to internalization in host cells. Thus, neutralization of HA in AI viruses by 3′-SL could eliminate viruses from birds by a cleansing mechanism, preventing their entry into the body.

The use of receptor analogs as inhibitors of adhesion of pathogens is a simple anti-adhesive method. In the presence of an excess amount of analog in the system, there will be competition between host cell surface receptors and analogs for binding with pathogens, which subsequently reduces the ‘true’ interaction between host cell receptors and pathogens, and also reduces the chances of developing diseases/infections^16^. Most microbial and animal interactions involve carbohydrate-containing receptors. Therefore, molecules mimicking these carbohydrate receptors are the main basis of anti-adhesion therapy^19^. In this study, we selected two SHMOs 3′-SL and 6′-SL as anti-adhesive molecules against several AI viruses in *in vitro* experiment. The *in vivo* study we carried out in chickens showed effective inhibition of H9N2 virus adhesion and invasion by 3′-SL, without developing any symptoms of flu and mortality of chickens after virus challenging.

## Results

### Hemagglutination inhibition (HI) assay of 3′-SL and 6′-SL against influenza A viruses

**Figure 1:**
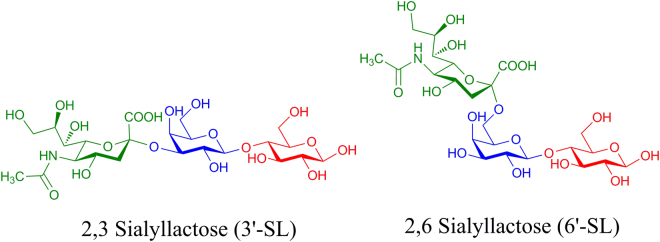
Sialylated human milk oligosaccharides. Structures of sialyllactose derivatives used in this study.

**Figure 2:**
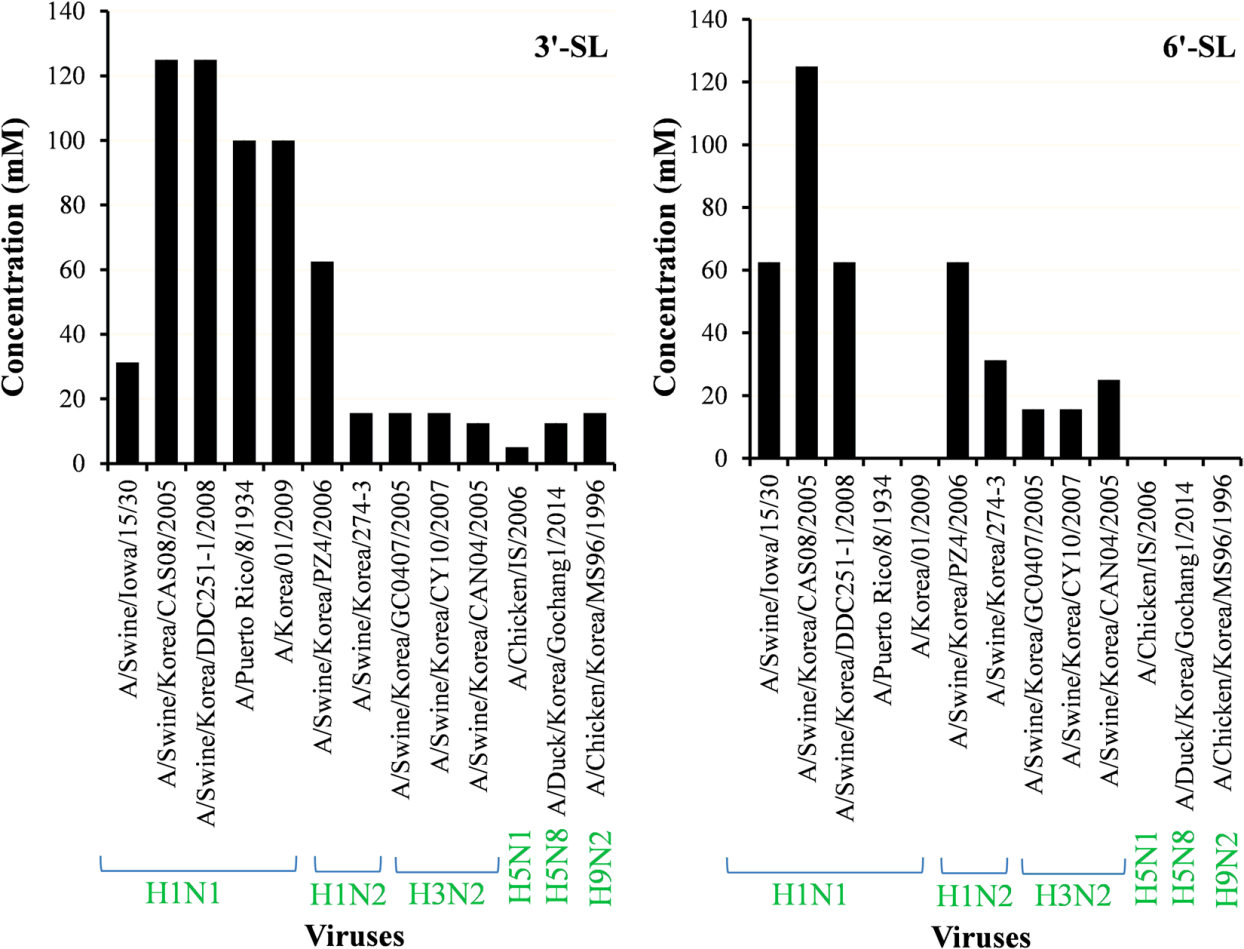
HI assay of 3′-SL and 6′-SL against various AI viruses. MIC values of 3′-SL and 6′-SL determined through HI assay against various subtypes of AI viruses. No values for two H1N1 strains A/Puerto Rico/8/1934 and A/Korea/01/2009, H5N1 strain A/Chicken/IS/2006, H5N8 strain A/Duck/Korea/Gochang1/2014, and H9N2 A/Chicken/Korea/MS96/1996 viruses with 6′-SL indicates MIC values of more than 200 mM. Data from triplicate assays for three independent experiments were plotted using SigmaPlot 10.0 from SYSTAT Software, Inc. San Jose, CA.

We assayed two derivatives of sialyllactose (3′-SL and 6′-SL) (Fig. 1) against thirteen different viruses included in six different subtypes (Table 1) in preliminary HI assay. The concept of HI assay using various concentrations of 3′-SL and 6′-SL studies the effectiveness of the prevention of binding of viruses to the chicken red blood cells (cRBCs) by SL derivatives. Sialic acid moiety of 3′-SL and 6′-SL binds to the hemagglutinin present in viruses. As a result, viruses are neutralized, hence preventing hemagglutinin mediated binding of viruses with sialic acids present in the surface of cRBCs. We determine the highest diluted concentration of SL-derivatives that prevents hemagglutination of cRBCs to be the minimum inhibitory concentration (MIC) (Fig. 2). 3′-SL showed promising results with all thirteen viral strains tested in this assay. Except H1N1 subtypes of viruses and one H1N2 subtype virus (A/Swine/Korea/PZ4/2006), MIC of 3′-SL was less than 16 mM with all the other viruses tested. In particular, the molecule showed the lowest MIC value of 5.0 mM with the highly pathogenic avian influenza (HPAI) strain of H5N1 subtype A/Chicken/IS/2006. Similarly, the MIC of another HPAI virus of H5N8 strain (A/Duck/Korea/Gochang1/2014) and one of the H3N2 subtype strains A/Swine/Korea/CAN04/2005 was 12.5 mM for 3′-SL. However, the MIC of H1N1 subtype viruses including (A/Swine/Korea/CAS08/2005, A/Swine/Korea/DDC-251-1/2008, A/Puerto Rico/8/1934, and A/Korea/01/2009 (Pandemic)) was 100 mM or higher (125 mM). Similarly, one of the H1N2 subtypes of viruses, A/Swine/Korea/PZ4/2006 also had a higher MIC value with 3′-SL. Other AI viruses H1N2 (A/Swine/Korea/274-3), H3N2 (A/Swine/Korea/GC0407/2005, A/Swine/Korea/CY10/2007), and H9N2 (A/Chicken/Korea/MS96/1996) had similar MIC values (15.62 mM) with 3′-SL (Fig. 2).

**Table 1:** Detail information of various avian influenza viruses used in this study.

No.	Subtype	Strain	Source
1	H1N1	A/Swine/Iowa/15/30	Animal and Palnt Quarantine Agency, S. Korea
2		A/Swine/Korea/CAS08/2005	
3		A/Swine/Korea/DDC-251-1/2008	
4		A/Puerto Rico/8/1934	
5		A/Korea/01/2009 (Pandemic)	
6	H1N2	A/Swine/Korea/PZ4/2006	
7		A/Swine/Korea/274-3	
8	H3N2	A/Swine/Korea/GC0407/2005	
9		A/Swine/Korea/CY10/2007	
10		A/Swine/Korea/CAN04/2005	
11	H5N1	A/Chicken/IS/2006 (HPAI)	
12	H5N8	A/Duck/Korea/Gochang1/2014 (HPAI)	
13	H9N2	A/Chicken/Korea/MS96/1996

We similarly determined the MIC value of another derivative of SL (6′-SL) against the same set of viruses. However, 6′-SL did not show MIC values against H1N1 (A/Puerto Rico/8/1934 and A/Korea/01/2009 strains), H5N1 (A/Chicken/IS/2006), H5N8 (A/Duck/Korea/Gochang1/2014), and H9N2 (A/Chicken/Korea/MS96/1996) subtype viruses at the concentration of as high as 200 mM. Although the result was positive with other subtype viruses, only H3N2 subtype group of viruses (A/Swine/Korea/GC0407/2005 and A/Swine/Korea/CY10/2007) had the lowest MIC value of 15.62 mM.

### *In vitro* anti-viral test of 3′-SL and 6′-SL against H9N2 using MDCK cell

**Figure 3:**
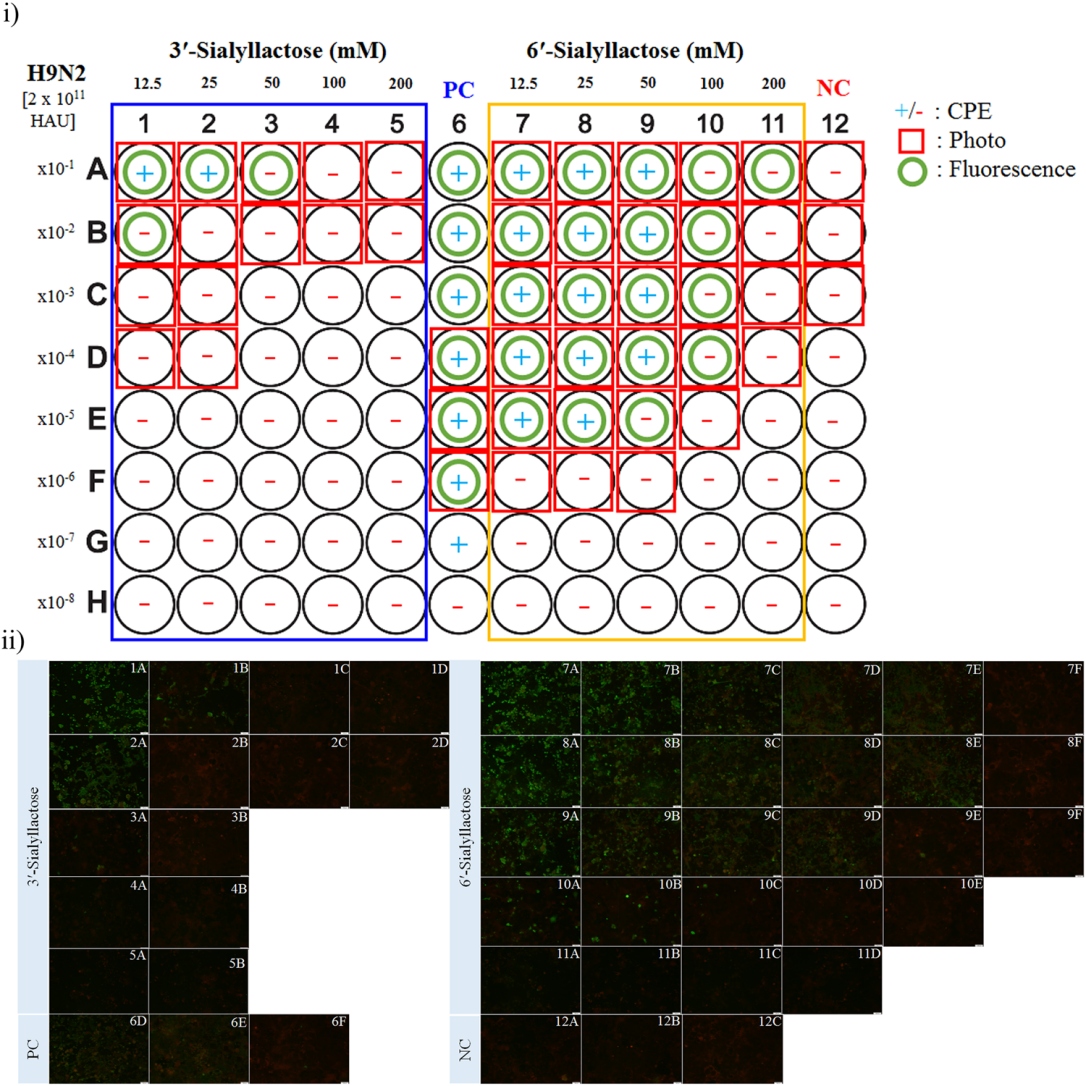
Experimental design and results of *in vitro* antiviral activity assay of 3′-SL and 6′-SL using MDCK cell line. (i) Experimental design. H9N2 (A/Chicken/Korea/MS96/1996) virus stock of 2 × 10^11^ HAU was serially diluted to x10^8^ folds. Each ten folds diluted virus sample was used to assay against different doses (12.5 to 200 mM) of 3′-SL and 6′-SL. The well shown within blue box (lane 1–5) were used for 3′-SL while wells within yellow box (lane 7–11) were used for 6′-SL. Each well in column of 3′-SL or 6′-SL containing different concentration of SHMOs were mixed with serially diluted H9N2. Lane 6 was positive control (PC) while lane 12 was negative control (NC). Plus (+) sign inside the well indicates cytopathic effect observed while minus (−) sign indicates non-cytopathic effect. Green circle well shows immunofluorescence staining while red square indicates photographs taken and presented. Minus (−) sign inside green circle indicates non-CPE but partial fluorescence observed in the photograph. (ii) Photograph of MDCK cells after immunostaining. Selected photographs are presented as indicated in experimental design (red circled wells) in Fig. 3(i).

To study the efficacy of 3′-SL and 6′-SL against H9N2 (A/Chicken/Korea/MS96/1996) virus *in vitro*, MDCK cell line was used and checked for cytopathic effect (CPE). Different number of viruses ranging from 2^11^ × 10^−1^ to 2^11^ × 10^−8^ HAU were mixed with 3′-SL and 6′-SL in dose dependent manner (12.5 mM to 200 mM) and used to infect approximately equal number of MDCK cells grown in 96 well plate as described in Methods. Positive experiment controls were kept without mixing viruses and either of the molecules, while negative control contained only MDCK cells and MEM medium. The detail experimental design is presented in Fig. 3(i). The summary of the outcome of the experiment is presented in Table 2 and supported by photographs of immunostained MDCK cells in Fig. 3(ii) to observe CPE and non-CPE cells during experiments.

**Table 2:** Summary of CPE of 3′-SL and 6′-SL neutralized different number of H9N2 (A/Chicken/Korea/MS96/1996) viruses along with positive control and negative control. Only 3′-SL and 6′-SL were also assayed for their CPE effect against MDCK cells as control experiments.

H9N2 (2^11^ HAU)	H9N2 + 3′-Sialyllactose (mM)					3′-Sialyllactose (mM)				H9N2 + 6′-Sialyllactose (mM)					6′-Sialyllactose (mM)				PC	NC
	12.5	25	50	100	200	25	50	100	200	12.5	25	50	100	200	25	50	100	200	H9N2	MDCK
x10^−1^	+	+	−	−	−	−	−	−	−	+	+	+	−	−	−	−	−	−	+	−
x10^−2^	+	−	−	−	−	−	−	−	−	+	+	+	−	−	−	−	−	−	+	−
x10^−3^	−	−	−	−	−	−	−	−	−	+	+	+	−	−	−	−	−	−	+	−
x10^−4^	−	−	−	−	−	−	−	−	−	+	+	+	−	−	−	−	−	−	+	−
x10^−5^	−	−	−	−	−	−	−	−	−	+	+	+	−	−	−	−	−	−	+	−
x10^−6^	−	−	−	−	−	−	−	−	−	−	−	−	−	−	−	−	−	−	+	−
x10^−7^	−	−	−	−	−	−	−	−	−	−	−	−	−	−	−	−	−	−	+	−
x10^−8^	−	−	−	−	−	−	−	−	−	−	−	−	−	−	−	−	−	−	+	−

The viral invasion of cell lead to lysis of the host cell or cell dies without cell lysis. Both of these phenomenon are due to CPE. The CPE was detected using two sets of antibodies as described in Methods. The virus infected cells were methanol fixed and treated with primary monoclonal antibody which is specific to nucleoprotein of H9N2 AI virus. The buffer washed cells were then visualized using anti-mouse fluorescein functionalized with an isothiocyanate reactive group (anti-mouse FITC) secondary antibody which gives green color upon binding with 3B19 antibody. Thus, the results were interpreted as green colored cells as virus lysed dead cells or CPE while non fluorescent cells as live cells or non-CPE.

When H9N2 stock was diluted ten and hundred times (2^11^ × 10^−1^ and 2^11^ × 10^−2^ HAU) and mixed with 12.5 mM of 3′-SL, CPE was observed (Fig. 3(ii) 1AB). But with more diluted virus (above x1000 of stock H9N2), no CPE was seen (Fig. 3(ii) 1CD). Similarly, CPE was seen when 25 mM of 3′-SL was mixed with only 2^11^ × 10^−1^ HAU viruses as shown in Fig. 3(ii) 2 A. No CPE was observed with further diluted viruses mixed with different concentration of 3′-SL. Importantly, when 3′-SL was used to treat MDCK cells to study the toxic effect, no cell death was observed with maximum of 200 mM of 3′-SL.

In contrast to 3′-SL, 12.5 mM, 25 mM, and 50 mM of 6′-SL was unable to neutralize ten thousand times diluted H9N2 stock (2^11^ × 10^−4^ HAU) viruses (Fig. 3(ii) 7A–E, 8A–E). But, further diluted viruses (above x10^5^ of stock H9N2) were neutralized as no CPE was seen (Fig. 3(ii) 7F, 8F). Further increased concentrations of 6′-SL (100 mM and 200 mM) equally neutralized H9N2 as 3′-SL did because no CPE was seen in remaining wells (Fig. 3(ii)10A–E and 11A–D).

This preliminary *in vitro* results showed that 3′-SL is effective to neutralize H9N2 viruses and prevented MDCK cell infection in comparison to 6′-SL at lower doses. These results were exciting to further study their possible potential applications in real control of AI virus outbreaks in the animal models. For a long time, South Korea’s poultry farming has faced a severe problem with H9N2 (A/Chicken/Korea/MS96/1996) infection. Thus, in this study, we selected 3′-SL among the two SL-derivatives, and focused our study on checking its effectiveness against the aforementioned Korean type H9N2 virus infection, using chicken as model animals by *in vivo* study.

### *In vivo* assay of 3′-SL against H9N2

Altogether, thirty male SPF chickens were used for the study. Among them, six chickens were kept as negative control (sub-group E), and were neither challenged with viruses nor treated with 3′-SL (Table 3). The experiment was designed in such a way that twenty-four chickens were divided into two major groups based on the virus load used to challenge them. The first group of twelve chickens (sub-group A and sub-group B) were challenged with 50 µL 8 HAU (10^6^ to 10^7^ 50% egg infective dose (EID_50_)/bird) of H9N2 viruses, whereas the second group of twelve chickens were challenged with a ten times lower number of viruses (50 µL 0.8 HAU; 10^5^ to 10^6^ EID_50_/bird of H9N2 viruses) (Table 3). We used half of the twelve chickens of both groups (sub-group B and D) as positive control (without treatment of 3′-SL), while the remaining six chickens of each group (sub-group A and C) were fed with 50 µL of 500 mM 3′-SL, as described in the Methods section. Oral and cloacal swabs of each chicken was collected every day until the ninth day, and analyzed by real-time polymerase chain reaction (qPCR) for the presence of virus. Moreover, we also monitored clinical signs and symptoms that developed on chickens.

**Table 3:** *In vivo* experimental design of 3′-SL with specific pathogen free chickens.

Chicken No.	Group	Sub-group	Sex	Virus Inoculation Material	3ʹ-SL Feeding after H9N2 inoculation
3	10^−2^ inoculation	Sub-group A (Control)	Male	Virus Only (50 µL 8 HAU + 50 µL D/W; RT 30 min mix)	Water−1 time a day D/W 1 mL for 9 days
7					
11					
12					
16					
23					
4		Sub-group B 3′-SL fed		Virus + 3′-SL Mixture (50 µL 8 HAU + 50 µL 500 mM SL; RT 30 min mix)	3′-SL−1 time a day 500 mM SL −1 mL for 9 days
13					
14					
15					
19					
21					
5	10^−3^ inoculation	Sub-group C (Control)		Virus Only (50 µL 0.8 HAU (1/10 dilution of 8 HAU) + 50 µL D/W; RT 30 min mix)	Water −1 time a day; D/W 1 mL for 9 days
9					
18					
20					
22					
24					
1		Sub-group D 3′-SL fed		Virus + 3′-SL Mixture (50 µL 0.8 HAU (1/10 dilution of 8 HAU) + 50 µL 500 mM SL; RT 30 min mix)	3′-SL−1 time a day 500 mM SL 1 mL for 9 days
2					
6					
8					
10					
17					
25	—	Sub-group E (Negative Control)		—	Water−1 time a day D/W 1 mL for 9 days
26					
27					
28					
29					
30					

**Figure 4:**
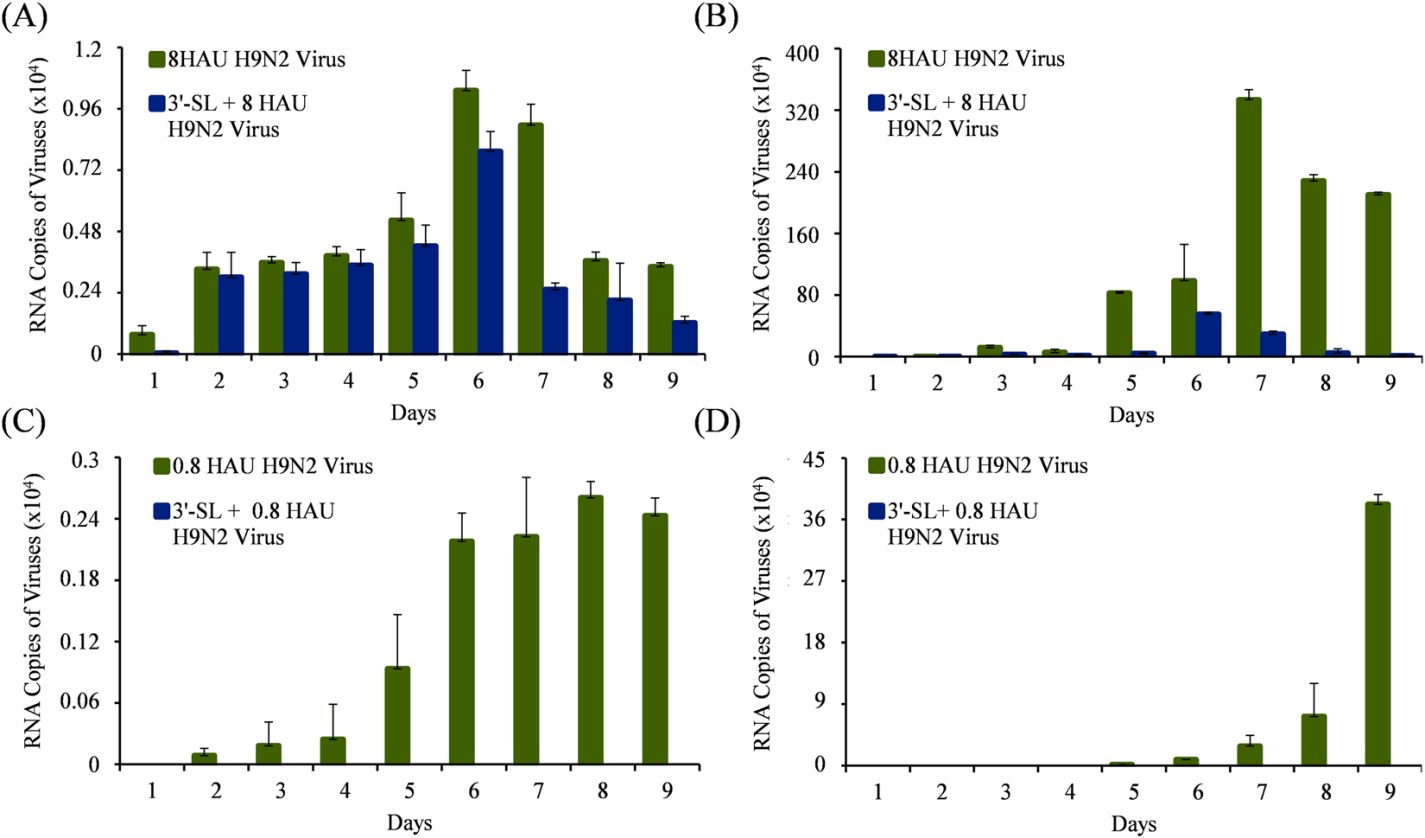
*In vivo* study of prevention of H9N2 infection by 3′-SL in SPF chickens. Number of virus count of sub-group A (8 HAU virus challenged) and sub-group B (8 HAU virus challenged and 3′-SL treated) chickens (**A**) oral swab and (**B**) cloacal swab. Number of virus count of sub-group C (0.8 HAU virus challenged) and sub-group D (0.8 HAU virus challenged and 3′-SL treated) chickens (**C**) oral swab and (**D**) cloacal swab. Sub-group E chickens were virus free chickens. Data from triplicate assays for three independent experiments were used to determine number of viruses in each sample from all sub-groups of chicken. Data were plotted using SigmaPlot 10.0 from SYSTAT Software, Inc. San Jose, CA. Data were reported as mean ± SEM.

The real time qPCR analysis of oral and cloacal swabs of each chicken was used for the determination of virus load on these samples. In 3′-SL untreated chickens (sub-group A and C), higher numbers of viruses were detected in both oral and cloacal swab samples than the respective 3′-SL treated chickens (Fig. 4). In comparison with sub-group A (8 HAU virus challenged), the number of viruses in both oral and cloacal swabs was lower in sub-group C (0.8 HAU viruses challenged) chickens. This is because of the lower initial viral challenge load. The virus shedding was found to be higher in oral swabs from the first day of challenge to the 9^th^ day, whereas the viral number started to increase in cloacal swabs in later days, such as after the 5^th^ day or 6^th^ day of a challenge (Fig. 4).

Oral swab of 3′-SL treated chickens challenged with 8 HAU of H9N2 (sub-group B) did not reveal any drastic decrease in number in comparison to the control chickens (sub-group A). However, we noticed reduction in the virus load in both oral and cloacal samples for each day. In cloacal samples of 3′-SL treated chickens; we detected a high load of viruses on the 6^th^ and 7^th^ days only, which decreased rapidly until the 9^th^ day (Fig. 4B). But when we treated 0.8 HAU viruses challenged chickens (sub-group D) with the same concentration of 3′-SL, the result was very encouraging. In both oral and cloacal swab samples of sub-group D chickens, no virus was detected each day from very beginning of the virus challenge till the 9^th^ day (Fig. 4C and D).

Interestingly, there was no virus, even on the first day of the virus challenge. This signifies that the inoculated virus was completely neutralized and washed away by 3′-SL within 24 h of the challenge (Fig. 4C and D). Since sub-group E chickens were SPF and antibody negative from the very beginning, they neither contained any viruses nor exhibited clinical symptoms, and survived throughout the experimental period.

We also monitored the flu symptoms, mortality, and weight loss of the chickens studied. None of the control chickens challenged with 8 HAU was found to be dead. Moreover, all chickens of each group survived throughout the experimental period. The 3′-SL treated, as well as positive and negative control chickens, did not develop any symptoms of flu, except for two chickens of sub-group A that showed facial edema; there was no loss in body weight.

### Serology

Enzyme-linked immunosorbent assay (ELISA) was performed to check the presence of antibody in the sera of the chickens used in this study, except for the chickens of sub-group E. We collected blood sample from each chicken at two-day intervals from the 4^th^ day of the virus challenge to the 12^th^ day for ELISA test.

**Figure 5:**
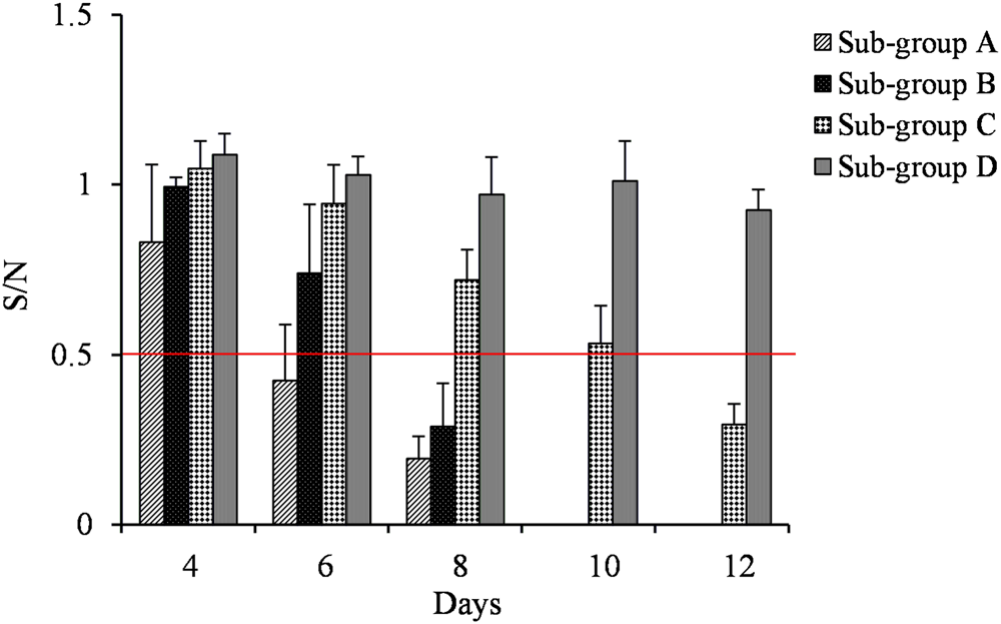
ELISA assay. ELISA assay of sera of four sub-groups of chicken in every two days interval from 4^th^ day to 12^th^ day. Sera of sub-group A and B were not assayed after 8^th^ day. Red line indicates cut off S/N value which is <0.5. Less than 0.50 indicates positive result (presence of antibody). Data from three triplicate assays for three independent experiments were plotted using SigmaPlot 10.0 from SYSTAT Software, Inc. San Jose, CA.

The results showed the presence of anti-H9N2 antibody in 8 HAU virus challenged chickens (sub-group A and B) on the 6^th^ day and 8^th^ day. Thus, we did not further analyze the sera of those chickens from the 10^th^ and 12^th^ days by ELISA. However, in the 0.8 HAU viruses challenged chickens (sub-group C), antibody was detected from the 8^th^ day onward (Fig. 5). The sample-to-negative (S/N) values were below cut-off value of 0.50 in all chickens within 12 days. We detected no antibody in 3′-SL treated chickens (sub-group D), as S/N values were higher than the cut-off value for all the tested chickens, confirming no entry of viruses into the blood stream of the chickens (Fig. 5). All the viruses in 3′-SL treated chickens of sub-group D were neutralized by 3′-SL; and as a result, they were unable to adhere and infect chickens.

## Discussion

Bird flu is a social problem in many countries, and causes severe economic and health problems. AI viruses are highly pathogenic to livestock, and also threaten human survival. Among many AI virus infections, H9N2 infection is an endemic outbreak in Korean poultry farms. It demonstrates clinical signs of depression, edema of head, cyanosis of comb and legs, and drop in egg production; and has a mortality rate of (5–30)%^20^. During the first outbreak of H9N2 in 1996, vaccination against AI viruses was not allowed. Later, its use was permitted in 2007, which efficiently prevented the spread of H9N2^21^. However, it was reported that Korean H9N2 subtype AI virus underwent antigenic drift by mutation at the antigenic sites, and could escape vaccine protection because of the excessive use of animal vaccination^22,23^. The consequence of antigenic drift could be new outbreaks of H9N2 in the near future. Thus, several vaccination strategies to animals are in practice to confront future outbreaks of H9N2 in Korea, and approaches are further advanced because of abundant genetic information on AI viruses. But vaccination against AI viruses has several issues related to the international trade of poultry and poultry products, and AI virus surveillance programs^23^. Thus, the search continues for a safe and effective alternative approach to control such infections and outbreaks, because in many cases, pre-existing vaccines and antiviral agents for the prevention and treatment of infections are not appropriate.

Recent studies on the binding affinity of AI A viruses hemagglutinin display preferences towards *α*2,3-linked sialyl glycans, while human influenza viruses are found to bind with *α*2,6-linked sialyl glycans^24–28^. Meanwhile, several studies have established the significance of SHMOs such as sialyllactose as a health beneficiary to humans, particularly infants, to gain resistance to microbial infections by neutralizing them, and preventing invasion^29,30^. Extensive studies have been carried out on sialyllactoses (3′-SL and 6′-SL) that compete with binding sites, and prevent the adhesion of pathogens such as *Salmonella*^31,32^, *Escherichia coli*^31–34^, *Helicobacter pylori*^32^, *Vibrio cholera*^31^, *Compylobacter jejuni*^32^ and rotaviruses^35^. Bacteria have been found to contain more than one adhesion protein in their surface. Moreover, other various factors also determine their adhesion to the receptors on the host cell surface, such as hydrophobic interactions, and non-specific interactions. Hence, preventing bacterial population from adhesion by a single anti-adhesion molecule is challenging and ineffective. Hence, multiple agents targeting each adhesion protein are necessary to effectively control pathogens by using anti-adhesion therapy^36,37^.

Previous experiments exhibited the reduced colonization of *H*. *pylori* in a mouse model when sialic acid was used in conjunction with catechins^38^. Similarly, other carbohydrate molecules *α*-methyl-galactoside and *α*-methyl-fucoside decreased mortality and health damage, after *P*. *aeruginosa* challenged lung injury^39^. The major drawback of the use of such sugar analogs is the requirement in higher dose to achieve bacterial adhesion inhibition, because of their low affinity with the target protein. In some instances, the problem has been addressed by linking with hydrophobic residues, which significantly increases the affinity of carbohydrate molecules with target proteins, lowering the concentration of analogs required for anti-adhesion^40–42^. However, the clinical trials of 3′-SL for the prevention of *H. pylori* in human^43^ and Rhesus monkeys^44^ and 3′-sialyllacto-*N*-neotetraose against *S. pneumoniae*, *Haemophilus infuenzae* and *Moraxella catarrhalis*^45^ were interrupted because of the use of multiple adhesins by these bacteria.

In this study, we observed broad spectrum neutralization of thirteen different viruses belonging to six different HA subtype viruses by both 3′-SL and 6′-SL. The efficacy of virus neutralization was different with different molecules in *in vitro* HI assay (Fig. 2). Interestingly, the MIC values were also different for the strains of the same HA subtype viruses. For example, among five H1N1 subtype strains, 3′-SL neutralized A/Swine/Iowa/15/30 efficiently (MIC = 31.25 mM) than other four strains for which MIC values were 100 mM or above. Likewise, among two strains of H1N2 subtype viruses, the MIC value for A/Swine/Korea/PZ4/2006 was higher (62.5 mM) than A/Swine/Korea/274-3 for which the value was 15.62 mM. The MIC values of three strains of H3N2 subtype viruses was also different. The similar pattern was observed in the case of 6′-SL with the same sets of viruses (Fig. 2). The possible reason for different antiviral efficacy by the same molecule against the same HA subtype virus strains could be because of the different receptor specificities of the HA of viruses^46^. Several previous studies established key role of HA present on viruses that account in part to determine avidity of receptor binding^24,26,46^. Thus, simple sequence analysis and homology modeling of HA does not always provide enough evidences to draw conclusions on receptor specificity of HA of strains belonging to the same HA subtype.

**Figure 6:**
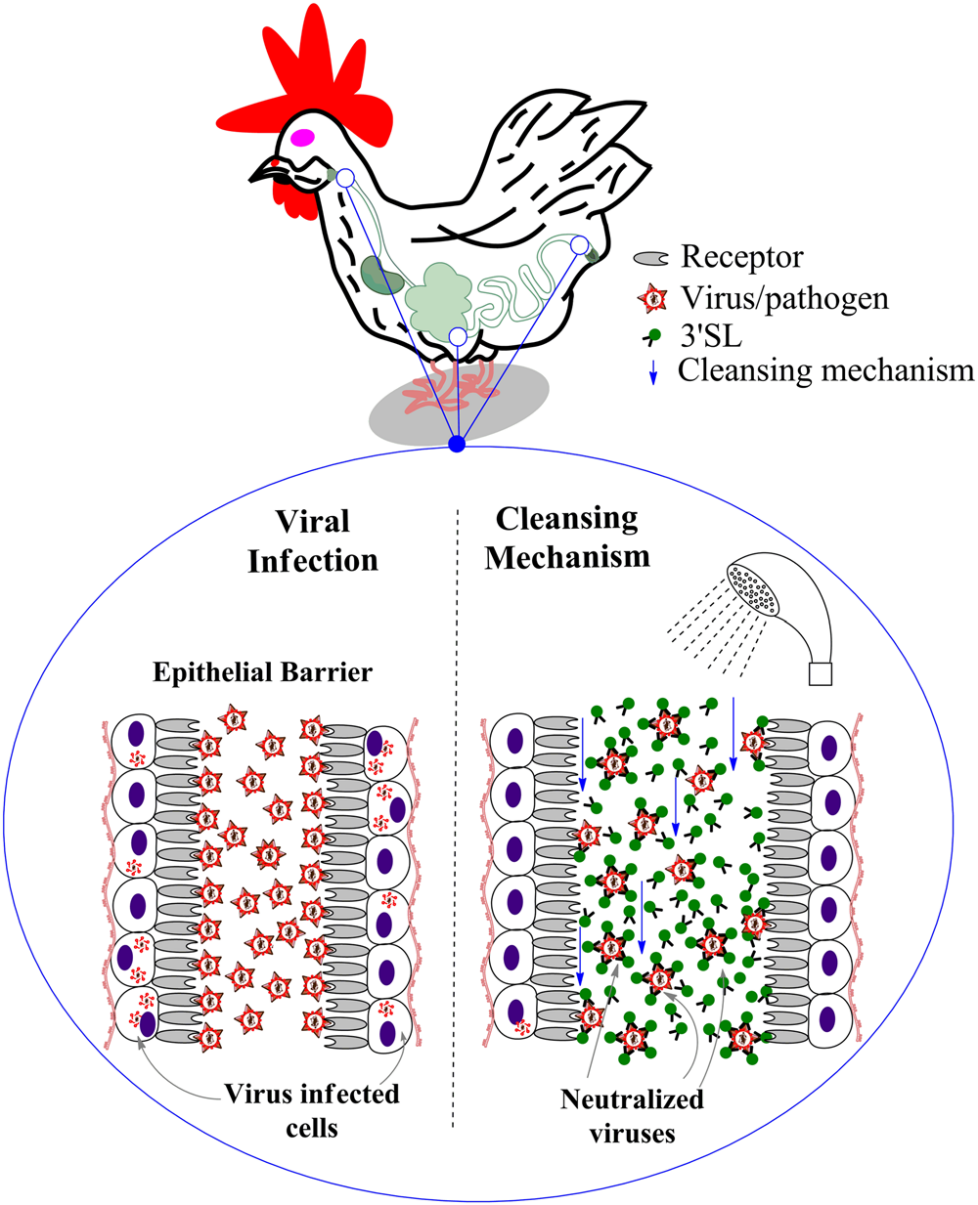
Cleansing mechanism. Neutralization of H9N2 viruses by 3′-SL and wash-out of viruses from the colon.

The same sialic acid containing molecule was applied for the control of H9N2 (A/Chicken/Korea/MS96/1996) virus infection in chickens for the first time. Since influenza virus uses haemagglutinin protein, a single receptor binding site for adhesion to host cells, it could be easy to neutralize and prevent infecting viruses from binding and invading host cell epithelia by generating competition on the binding site by feeding receptor analogs, as Fig. 6 depicts. The mechanism of preventing pathogens for adhesion to host cell receptors and invasion of host cell during infection cycle; and finally eliminating them out of the body by simple wash out is well established as cleansing mechanism (Fig. 6). This mechanism is also naturally present in animal’s immune defense system. 3′-SL mimicked as host cell receptors of epithelial cell to H9N2 virus which preferentially bind to *α*2,3-linked sialyl glycans present in avian mucus cells. Thus, 3′-SL effectively neutralized H9N2 virus by binding at its receptor binding sites and apparently removing them from colon by simple wash out (Figs 4 and 6). Interestingly, when 0.8 HAU of viruses were used to challenge the chickens and treated with 3′-SL every day, the virus load was nil within 24 hours. There were no observable clinical signs, symptoms, or side effects in all chickens receiving 3′-SL. ELISA assay of the sera of the chickens also confirmed the complete prevention of virus entry into the blood stream, since we detected no antibody against H9N2 virus. Viruses used for infection were completely neutralized by 3′-SL and excreted out of the body within 24 hours, as we detected no virus in the oral and cloacal swab samples (Fig. 4). Thus, this molecule could be a promising compound for the prevention of H9N2 virus infection in chickens. Although anti-adhesion therapy needs a higher dose of non-toxic molecules, multimers of 3′-SL containing molecules will certainly address this problem, and lower the use of 3′-SL concentration to prevent virus adhesion. On the other hand, 3′-SL is a non-toxic molecule that is naturally present in human milk. It also did not exhibit toxic effect at 200 mM in MDCK cells in *in vitro* experiment (Table 2). Thus, there is no doubt on the toxicity issue of this molecule, which is completely safe to humans and other animals.

Interestingly, we observed relatively low concentration of 3′-SL effectively inhibited two HPAI strains H5N1 (A/Chicken/IS/2006) and H5N8 (A/Duck/Korea/Gochang1/2014) in hemagglutinin inhibition assay with MIC of 5 mM and 12.5 mM, respectively (Fig. 2). This result opens up the possible path for future research works on 3′-SL to prevent these HPAI strains to cause outbreaks which results in causalities, economic burden and threats to public health in the society. Further research works on the same molecule to extensive level could aid better controlling means of recent outbreak of H5N8 in South Korea which raised the nation’s bird flu alert to its highest level banning the movement of farmers and transportation of poultry and poultry products. Moreover, the same outbreak dramatically raised local market prices of eggs and poultry products.

This preliminary *in vivo* study of 3′-SL against H9N2 (A/Chicken/Korea/MS96/1996) infection in chicken needs further extensive study, prior to use at an industrial level. Moreover, clinical study of this molecule to use as anti-adhesive drug for the treatment and prevention of viral infections is obligatory.

## Methods

### Compounds

Sodium salt of 3′-sialyllactose (3′-SL; Neu5Ac*α*2-3Gal*β*1-4Glc*β*; empirical formula: C_23_H_39_NO_19_; molecular weight: 633.55) and sodium salt of 6′-sialyllactose (6′-SL; Neu5Ac*α*2-6Gal*β*1-4Glc*β*; empirical formula: C_23_H_39_NO_19_; molecular weight: 633.55) were obtained from GeneChem Inc (Daejeon, South Korea). Figure 1 presents the structures of both molecules used in this study.

### Medium and Reagents

Minimal essential medium (MEM) was purchased from Gibco^TM^ (Thermo Fisher Scientific, Seoul, Korea). Monoclonal antibody (3B19) to AI virus nucleoprotein prepared using H9N2 strain as immunogen was purchased from Median Life Science (South Korea). Anti-mouse –FITC secondary antibody was purchased from (Thermo Fisher Scientific, Seoul, Korea).

### Viruses and Cell Line

Altogether, we used thirteen different viruses of six different subtypes of AI viruses for *in vitro* antiviral activity test using HI assay. Five subtypes of H1N1, two subtypes of H1N2, three subtypes of H3N2, a H5N1, a H5N8, and a H9N2 (Table 1) were used for preliminary *in vitro* analysis of antiviral activity of 3′-SL and 6′-SL. All those strains were obtained from the Animal and Plant Quarantine Agency (http://www.qia.go.kr/, S. Korea). For *in vivo* experiment and other use, we propagated H9N2 strain in 10-day-old SPF chicken eggs, and maintained them at −70 °C as stock. To challenge chickens, stock of virus was melted and directly used it. All experiments were performed using a biosafety level-2 (BSL-2) facility. Madin Darby Canine Kidney cell line (MDCK) (PTA-6500) was used for *in vitro* studies.

### Chickens

Six-weeks-old SPF chickens were purchased from NamDuck SPF (Korea). All experiments were conducted in accordance with relevant guidelines and regulations for experimental animal use approved by the Animal Ethics Committee of the Animal and Plant Quarantine Agency of Korea. Chickens were maintained under standard laboratory conditions in a chicken isolator.

### Hemagglutination inhibition (HI) assay

The HI assay was slightly modified and performed as described in the OIE Terrestrial Manual^47^. We added 12.5 µL of 3′-SL and 6′-SL at the indicated concentrations to 96-well plates by serial dilution. 12.5 µL of 4 hemagglutination units (HAU) of virus antigen (4HAU/12.5 µL) was added to the wells. The reaction was incubated at room temperature for 30 minutes. Finally, 25 µL of 1% (V/V) chicken red blood cells (cRBCs) was added, mixed gently, and continued to incubate at room temperature for 40 minutes to settle down cRBCs. The wells were visually inspected for the presence or absence of hemagglutination. Positive and negative controls without treatment or without viruses were also included to analyze the result. All assays were performed in triplicate. We determined the HI titre to be the highest dilution of compound causing complete inhibition of 4 HAU of virus antigen.

### *In vitro* antiviral activity assay using MDCK cell line

MDCK cells were cultured in a 96-well cell culture plate at a concentration of 2 × 10^5^ cells/mL for 18 to 24 hours before the experiment. Experiment was started when 80–90% cell were grown. In new 96 well plate, 50 µL of sialyllactose (3′-SL and 6′-SL, separately) was added at following different concentrations 25 mM, 50 mM, 100 mM, 200 mM, and 400 mM. To this plate, appropriately diluted 50 µL of virus was added from 2 × 10^11^ HAU virus stock. Sialyllactoses were diluted two folds making final concentration of (12.5 mM, 25 mM, 50 mM, 100 mM, and 200 mM). In the case of positive control, 1: 1 ratio of mixture of virus and MEM was added without SL. For negative control, 100 µL of MEM was added. It lacked virus as well as SL. The plate was kept at 37 °C for 1 hr. From the MDCK cell culture wells, medium was removed. To this cell, mixture of virus and SL was added. After 2–4 days of incubation CPE was detected.

### Immunofluorescence protocol

After 2–4 days of incubation for CPE study, the supernatant was removed. To each well 100 μL cold methanol was added and allowed to dry at room temperature for 20 minutes. The first monoclonal antibody (3B19; Nucleo Protein target) was dispensed at 100 μL per well and reacted at 37 °C for 1 hour. From this plate, the supernatant was removed and washed three times with phosphate buffer saline (PBS). Then, the second antibody 100 µL (Anti-mouse FITC) was added to each well and kept at 37 °C for another 1 h. Supernatant was removed and washed three times with PBS and checked the fluorescence and photographs were taken.

### *In vivo* experimental design

Six-weeks-old SPF chickens were housed in the chicken isolator (model No. SK-ISO-600 HBC2 (Three-Shine Inc., Korea) for three days prior to *in vivo* experiments. Thirty SPF chickens were divided into five sub-groups (A to E), each containing six experimental chickens (Table 2). The first two sub-groups (A and B) were challenged with 50 µL of 8 HAU H9N2 viruses, another two sub-groups (C and D) were challenged with 50 µL of 0.8 HAU of H9N2 viruses, while sub-group E chickens were neither virus challenged, nor treated with 3′-SL (negative control). One from each two-virus challenged groups (sub-groups A and C) were kept as positive control without feeding 3′-SL, while another two sub-groups (B and D) were fed with 500 mM 3′-SL. Prior to challenge of sub-groups B and D chickens, 50 µL of H9N2 viruses (8 HAU and 0.8 HAU) were mixed with 50 µL of 500 mM 3′-SL. For the following nine days, 1 mL of 500 mM 3′-SL was fed one time a day to both sub-group B and D chickens, while control chickens (sub-groups A, C and E) were fed with the same volume of sterile water (Table-2). The virus challenge and 3′-SL feeding were done intranasally.

Prior to virus challenge, the sera of all SPF chickens were collected for immunoassay to identify the antibodies against nucleoproteins of influenza A (specifically H9N2 subtype) by using commercial ELISA kits.

All the chickens were monitored daily for 9 days for clinical signs, symptoms of diseases and mortality after the virus challenge. At intervals of two days, 1 mL of blood sample was collected from all challenged chickens (sub-group A to D), and the sera stored at −70 °C for further study. The cloacal and oral samples were also collected every day, and stored at −70 °C, until further use. The weight of cloacal and oral samples was measured for the exact dilution rate to perform real time qPCR for quantification of the excreted viruses.

### Viral RNA preparation and Quantitative reverse transcriptase polymerase chain reaction (qRT-PCR)

Virus RNA was extracted from 150 µL of the oral and cloacal swab samples using the RNeasy® Mini Kit (QIAGEN) according to the manufacturer’s recommendation, and eluted in 50 µL of RNase-free water. Real-time qPCR was carried out with the WizPure^TM^ qPCR 2× Master Kit (Wizbio, Korea) with the Bio-rad CFX96 system (Bio-rad) in a 20 µL mixture containing 5 µL total RNA, 10 µL 2× qRT-PCR Master mix, 0.2 µM forward primer, 0.2 µM reverse primer and 0.2 µM probe^48^. The reaction was performed for 30 min at 50 °C, followed by 5 min at 95 °C, with a subsequent 40 cycles of amplification (95 °C for 10 s, 55 °C for 30 s, 72 °C for 10 s). Fluorescence was recorded at 55 °C. The primer sequence used were M + 25 (5′-AGATGAGTCTTCTAACCGAGGTCG-3′), M-124 (5′-TGCAAA**A**ACA**TC**TTC**A**AGTCTCTG-3′), and M + 64 (5′-FAM-TCAGGCCCCCTCAAAGCCGA-TAMRA).

Serial dilutions of the *in vitro*-transcribed RNA ranging from 1 × 10^7^ to 1 copies/µL were used to perform the sensitivity test. RNA was *in vitro*-transcribed from a recombinant pGEM^®^T plasmid (Promega) containing a synthetic 101 bp (target size) M gene of the H9N2 virus (A/Korea/MS96/1996) using the RiboMAX^TM^ Large Scale RNA Production System-T7 (Promega), following the manufacturer’s recommendations. The sensitivity of the H9N2 assay was 5 copies of target RNA per reaction.

### Enzyme-linked Immunosorbent assay (ELISA)

ELISA assay kit (IDEXX Influenza A Ab test kit, IDEXX Laboratories, Inc (USA)) was used to determine the relative level of antibody against H9N2 virus in challenged animals’ serum. The assay was performed on 96-well plates that had been coated with influenza A antigen. The sera were ten times diluted with the dilution buffer prior to ELISA assay. The antigen coated plates were obtained, and the position of different samples along with control samples was noted on a worksheet. In two wells, 100 µL of undiluted negative control was dispensed, whereas the same volume of undiluted positive control was dispensed in another two wells. In the remaining wells, 100 µL of diluted samples were added and incubated for an hour at (18–26) °C. Wells were washed with approximately 350 µL of wash solution for 3–5 times. Thereafter, 100 µL of conjugate was added into each well, and incubated for 30 minutes at 18–26 °C. Then, all wells were gently washed with wash solution 3–5 times. 100 µL of 3,3′,5,5′-Tetramethylbenzidine substrate solution was added to all the washed wells, and incubated at 18–26 °C for the next 15 minutes. We recorded the absorbance of all samples along with positive and negative control samples at 650 nm, then calculated the results using the following formula:

$$\begin{array}{rcl}{\rm{Negative}}\,{\rm{control}}\,{\rm{mean}}\,(\mathrm{NC}\overline{{\rm{x}}}) & = & ({{\rm{NC}}}_{1}{{\rm{A}}}_{650}+{{\rm{NC}}}_{2}{{\rm{A}}}_{650})/2\\ {\rm{Negative}}\,{\rm{control}}\,{\rm{mean}}\,({\rm{NC}}\overline{{\rm{x}}}) & = & ({{\rm{NC}}}_{1}{{\rm{A}}}_{650}+{{\rm{NC}}}_{2}{{\rm{A}}}_{650})/2\\ {\rm{Test}}\,{\rm{sample}}\,({\rm{S}}{\rm{/}}{\rm{N}}) & = & {\rm{Sample}}\,{\rm{mean}}\,{{\rm{A}}}_{650}/\mathrm{NC}\overline{{\rm{x}}}\end{array}$$
